# Detection and whole genome sequence analysis of an enterovirus 68 cluster

**DOI:** 10.1186/1743-422X-10-103

**Published:** 2013-04-02

**Authors:** Angela K Todd, Richard J Hall, Jing Wang, Mathew Peacey, Sharla McTavish, Christy J Rand, Jo-Ann Stanton, Susan Taylor, Q Sue Huang

**Affiliations:** 1 Institute of Environmental Science and Research Limited, National Centre for Biosecurity and Infectious Disease, 66 Ward Street, Wallaceville, Upper Hutt, 5018, New Zealand; 2 Department of Anatomy, University of Otago, P. O. Box 913, Dunedin, 9054, New Zealand; 3 Middlemore Hospital, Otahuhu, Auckland, 2025, New Zealand

**Keywords:** Enterovirus 68, Respiratory tract infections, Molecular genome sequencing

## Abstract

**Background:**

Enteroviruses are a common cause of human disease and are associated with a wide range of clinical manifestations. Enterovirus 68 is rarely detected yet was reported in many countries in 2010. Here enterovirus 68 was identified for the first time in New Zealand in 2010 and was detected in a further fourteen specimens over a six month period.

**Objectives:**

To genetically characterise enterovirus 68 specimens identified in New Zealand in 2010.

**Study design:**

The genome sequence of a New Zealand representative enterovirus 68 isolate was obtained. Ten clinical specimens were analysed by sequencing the VP1 region of the enterovirus 68 genome.

**Results:**

Based on sequence analysis of the VP1 region and the full genome of one representative isolate, the New Zealand enterovirus 68 isolates clustered with contemporary enterovirus 68 viruses and do not show any clear distinguishing genetic diversity when compared to other strains. All fifteen specimens showed high similarity with enterovirus 68 by VP1 sequencing. The majority of New Zealand patients suffered from bronchiolitis, were less than two years of age and were of Pacific Island or Maori descent.

**Conclusions:**

We document the rare occurrence of an enterovirus 68 cluster in New Zealand in 2010. These viruses shared similarity with other clusters of enterovirus 68 that occurred globally in 2010. A greater awareness in enterovirus 68 infection may help detect this virus with increased frequency and enable us to better understand the role this strain plays in disease and the reasons behind this global emergence in 2010.

## Background

Enteroviruses belong to the *Picornaviridae* family and are among the most commonly identified aetiological agents of human disease [1]. There are approximately 100 enterovirus serotypes which cause a range of clinical manifestations; from asymptomatic infections to more serious illnesses such as aseptic meningitis, myocarditis and acute flaccid paralysis [1,2]. Enterovirus 68 (EV68) is a member of the Human enterovirus D species and was first isolated in California, USA in 1962 from children who were hospitalised with lower respiratory tract infections [3]. Since then, EV68 has been isolated rarely; only 26 strains have been identified over 36 years in the USA [2]. EV68 is unique among enteroviruses in that it has a lower than optimum growth temperature and is acid sensitive [4-6]. As such, it shares characteristics with human rhinovirus [4,5]. It is further unique in that it is almost exclusively associated with respiratory disease [4,5]. Recently EV68 has been isolated with increased frequency. Its isolation has been reported in Germany, the Philippines, Thailand, Italy, Japan, the United States, the United Kingdom and the Netherlands with the majority of these reports occurring in 2010 [7-15]. To date, only four full genome sequences of EV68 have been published; that of the prototype Fermon strain, the French 37–99 strain and two EV68 strains that were circulating in Japan in 2010 [5,9,16].

## Objectives

In the current study we describe fifteen cases of EV68 isolated from samples taken from March to August 2010 in New Zealand. All cases were initially identified by partial VP1 sequencing. Due to limiting sample volumes complete VP1 sequencing was performed on only ten of the fifteen EV68 samples confirming EV68 infection. Additionally, characterisation of the full genome sequence of a representative New Zealand EV68 isolate was achieved by Roche 454 sequencing.

## Study design

### Patients and specimen collection

The National Poliovirus and Enterovirus Identification Reference Laboratory at the Institute of Environmental Science and Research Limited, National Centre for Biosecurity and Infectious Disease routinely receives untyped enterovirus clinical specimens or cell culture isolates from four major hospitals (based in Auckland, Waikato, Wellington and Christchurch) as part of the New Zealand enterovirus surveillance network.

### Viruses and cells

Human rhabdomyosarcoma (RD) cells (passage 242–256) were propagated in 10% Hanks Minimal Essential Medium (Gibco, Life Technologies, Carlsbad, CA, USA) supplemented with 10% (v/v) foetal bovine serum (HyClone, New Zealand), 7.5% (v/v) sodium bicarbonate, 1% (v/v) 1 M hepes and antibiotics.

### RNA extractions

Viral nucleic acid was extracted (400 μL of clinical specimen or cell culture isolate) using the Zymo ZR Viral RNA Kit™ (Zymo Research Corporation, Irvine, CA, USA) as per the manufacturer’s instructions. Nucleic acid extracts (20 μL) were stored at −80°C until required.

### Partial and full VP1 RT-PCR and sequencing

Initially, specimens were characterised by amplification and sequencing of a 375 bp partial VP1 region as described previously [17]. An EV68 RT-PCR assay was designed in-house using Primer3 v 0.4.0 [18] in order to specifically amplify and sequence the entire VP1 region. The forward primer was designated EV68-VP1-Forward (5’-GCA-GCC-TAT-CAG-GTG-GAG-AG-3’) and the reverse primer was designated EV68-VP1-Reverse (5’-TGC- TCA-TGT-ATG-GCA-TGG-TT-3’) resulting in a product that was 805 bp in length. One-step RT-PCR assays were performed using the SuperScript III Platinum One-Step System (Invitrogen, Life Technologies). Each 50 μL reaction contained 5 μL of RNA, 25 μL of 2X Reaction Mix, 0.5 μL of reverse transcriptase-*Taq* polymerase enzyme mix, 20 units of RNase inhibitor(Invitrogen) and 0.2 μM of primers. Following an initial 50 minute reverse transcription at 50°C and 15 minute denaturation step at 94°C, a three-step cycling procedure of denaturation at 94°C for 30 seconds, annealing at 55°C for 30 seconds and extension at 72°C for 1 minute over 40 cycles was used. A final extension was performed at 72°C for 5 minutes. Each assay included negative and positive extraction controls and RNase-free reagents and procedures were used to minimise contamination.

Samples were run on an E-Gel® EX 2% precast agarose gel (Invitrogen). Bands of interest were purified using the Qiagen QIAquick PCR Purification Kit (Qiagen Incorporated, Valencia, CA, USA) and used as templates for sequencing on an ABI 3130XL automated DNA sequence using ABI Big Dye v3.1 technology (Applied Biosystems, Life Technologies).

### Phylogenetic analysis

Sequences were edited and assembled using Bionumerics 5.10 (Applied Maths, Belgium) and compared to known VP1 sequences in GenBank using BLAST [19]. Samples were determined to be EV68 strains if they were ≥ 75% similar to the prototype EV68 Fermon strain (GenBank accession number: AY426531).

The phylogenetic relationships were inferred using the Neighbour-Joining method (Tamura 3-parameter model with gamma distributed rates among sites) on nucleotide sequence data from the VP1 region, incorporating 651 base pairs. All positions containing gaps and missing data were eliminated. Evolutionary analyses were conducted in MEGA5 [20] (Center for Evolutionary Medicine and Informatics, Tempe, AZ, USA). 1000 bootstrap replicates were performed to determine the consensus tree.

### Enterovirus genome sequencing

Full-length genomic sequence of EV68 was obtained by sequencing on a Roche GS FLX instrument. Passage 1 viral culture was firstly purified to enrich for virus particles. A 1 mL volume of viral culture was subjected to three cycles of freeze-thaw lysis between −80°C and 37°C and then to centrifugation at 3000 × *g* for 15 minutes. To remove cellular RNA, 400 μL of supernatant was treated with RNAse A (Ambion, Life Technologies) at a final concentration of 10 μg.mL ^-1^ at 37°C for one hour. RNA was extracted from this fraction using the Ambion MagMax Viral RNA kit (Ambion) with elution into 40 μL of EB buffer. The RNA concentration was not high enough for direct application to the Roche GS FLX sequencer so a random amplification method for low yield samples was used, as has been described previously [21]. 500 ng of this material was used to make a barcoded, GS FLX Rapid library (Roche, Mannheim, Germany) according to the manufacturer’s instructions except that the sample was not nebulized. Completed library length was between 500 bp and 900 bp and the concentration was 8.35 × 10^9^ molecules/μL. Two emulsion PCR reactions were performed using a library concentration of 4 counts per bead and the enriched beads combined to give a total of 137,200. These were loaded onto a 1/16th region of a pico titre plate and sequenced using the XLR70 Titanium chemistry (Roche). The utilities sffinfo [22] and FASTX-Toolkit [23] were used to remove adaptor and primer sequences and reads filtered based upon data quality. This also increased BLAST search performance because redundant sequence information was not submitted. Human EV68 sequence was assembled using a reference strain, Fermon (GenBank accession number: AY426531). Assembly and annotation was achieved using Geneious 5.5.6 (Biomatters Ltd., New Zealand), on enterovirus hits obtained from the BLASTN search. Areas of assembly with less than 5X coverage were resequenced by the sanger-sequencing methodology using custom designed PCR primers.

## Results

### Patients and clinical data

In 2010, EV68 was detected in a total of fifteen samples. EV68 positive patients ranged in age from 1 month to 48 years, median age 11 years and 0 months. The majority of patients (*n* = 11; 73.3%) were less than 2 years of age. Patients testing positive for EV68 were predominantly male (*n* = 10; 66.7%) and most were identified as being of Pacific Island or Maori descent (*n* = 12; 80.0%) (Table 1). The first EV68 positive sample was taken in March of 2010. The peak incidence was observed in June of 2010 (*n* = 7; 46.7%) and this coincides with the winter period in New Zealand, typical of respiratory disease. Most of the samples originated in the Auckland region (n = 13; 86.7%) with the remaining two samples coming from the Waikato region. Respiratory samples included throat swabs (*n* = 1; 6.7%), nasopharyngeal swabs (NPS) (*n* = 4; 26.7%), nasopharyngeal aspirates (NPA) (*n* = 9; 60.0%) and a tracheal aspirate (*n* = 1; 6.7%). Patients presented with bronchiolitis (*n* = 6; 40.0%), asthma (*n* = 2; 13.3%) or other symptoms including cough, stridor, coryza and wheezing (Table 1).

**Table 1:** Characteristics of fifteen patients in whom enterovirus 68 was isolated in New Zealand in 2010

**Patient**	**Accession number**	**VP1 sequence**	**Strain name**	**Age**	**Sex**	**Ethnicity**	**Sample taken**	**Sample type**	**Health district**	**Clinical details**
1	JQ13905	Partial	NZ-2010-183	5 m	M	Maori	March	Nasal	Waikato	Bronchiolitis
2	JQ13906	Partial	NZ-2010-358	14 m	M	Maori	May	Tracheal Aspirate	Auckland	Burns
3	JQ13907	Partial	NZ-2010-404	6 m	M	Other	May	NPA	Auckland	Bronchiolitis
4	JQ13908	Partial	NZ-2010-431	18 m	F	Other	June	NPA	Auckland	Cough, Stridor, Wheeze
5	JQ13909	Partial	NZ-2010-435	5 m	M	Pacific Island	June	NPA	Auckland	Cough, Coryza
6	n/a	No	NZ-2010-475	15 m	M	Maori	June	NPA	Auckland	Cough, Coryza, Wheeze
7	JQ13910	Partial	NZ-2010-481	26 y	F	Pacific Island	June	NPS	Auckland	Pertussis
8	JQ13911	Partial	NZ-2010-539	12 m	M	Pacific Island	June	NPA	Auckland	Severe asthma
9	JQ13912	Complete	NZ-2010-541	7 m	M	Pacific Island	June	NPA	Auckland	Bronchiolitis
10	JQ13913	Partial	NZ-2010-571	1 m	M	Pacific Island	June	NPA	Auckland	Cough, Coryza
11	n/a	No	NZ-2010-580	46 y	F	Maori	July	NPS	Auckland	Sepsis, Heart Failure
12	n/a	No	NZ-2010-594	2 m	M	Pacific Island	July	NPA	Auckland	Bronchiolitis
13	n/a	No	NZ-2010-734	38 y	F	Maori	July	NPS	Auckland	Asthma
14	n/a	No	NZ-2010-1058	4 m	M	Cook Island Maori	August	NPA	Auckland	Bronchiolitis
15	JQ13904	Partial	NZ-2010-1507	48 y	F	Unknown	August	Throat	Waikato	Bronchiolitis

*m* = months; y = years; *M* = male; *F* = female; *NPA* = nasopharyngeal aspirate; *NPS* = nasopharyngeal swab.

### VP1 Phylogenetic analysis

**Figure 1:**
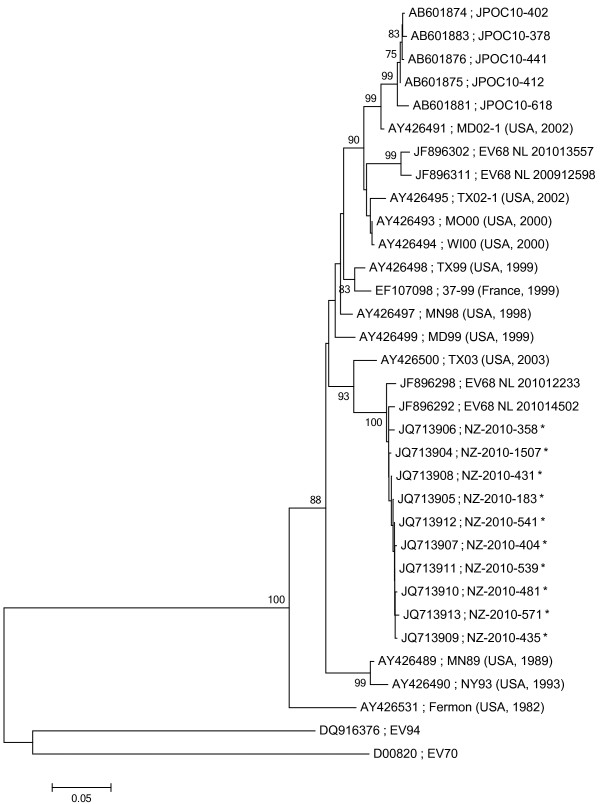
**The evolutionary history was inferred using the Neighbour-Joining method (Tamura 3-parameter model with gamma distributed rates among sites) on nucleotide sequence data from the VP1 region, incorporating 651 base pairs.** All positions containing gaps and missing data were eliminated. Evolutionary analyses were conducted in MEGA5^20^ (Center for Evolutionary Medicine and Informatics, Tempe, AZ, USA). 1000 bootstrap replicates were performed to determine the consensus tree presented in this figure, support for nodes present in greater than 75% of the trees are annotated. The viruses detected in this study are marked with an asterisk. The GenBank accession number and the strain designation is shown for each virus.

Based upon sequencing of the VP1 gene segment of ten New Zealand clinical isolates, the viruses grouped in one distinct phylogenetic cluster (Figure 1). Enteroviruses were judged to be the same serotype if they were ≥ 75% similar to the prototype Fermon strain (GenBank accession number: AY426531) but had < 70% sequence similarity to any other reference strain [17]. The ten New Zealand EV68 isolates had an average of 88.6% similarity to the VP1 of the prototype Fermon strain and only 66.5-68.8% similarity to that of enterovirus 70 (GenBank accession number: DQ201177) and 94 (GenBank accession number: DQ916376) (other species D enteroviruses), thus confirming these viruses as EV68. The New Zealand isolates were 98.0-99.5% identical to each other. The New Zealand EV68 VP1 nucleotide sequences were compared with the sequences of EV68 from the Philippines, Japan and the Netherlands. The VP1 phylogenetic tree demonstrated that the New Zealand EV68 isolates clustered most closely with EV68 isolates that were detected in the Netherlands in 2010 but were not sufficiently distinct from any other EV68 sequence so as to identify them as a genetically distinct group.

### Enterovirus whole genome sequencing

We describe the fifth published complete genome of an EV68 virus with the exception of 32 nucleotides in the 5’ UTR which could not be determined either by high throughput or sanger sequencing. The Fermon (GenBank accession number: AY426531), 37–99 (GenBank accession number: EF107098) and two Japanese strains (GenBank accession numbers: AB601882 and AB601883) are the only EV68 strains for which complete genome sequences are available. High-throughput sequencing of NZ-2010-541 (passage 1, viral culture) on 1/16th of a Roche GS FLX Titanium plate produced 41,349 pass filter reads. 40,091 reads were identified as human enterovirus by a BLASTN search of the GenBank non-redundant nucleotide database. 26,523 high quality reads were used to assemble a genome sequence for NZ-2010-541 of 7,320 bp (GenBank accession number: JX070222). The closest complete genome sequence identified was that of the strain 37–99 (GenBank accession number: EF107098) which showed 93.40% nucleotide sequence identity overall (Table 2). The genome sequence of NZ-2010-541 is unremarkable when compared to the other published genomes and no evidence for recombination was revealed.

**Table 2:** Pairwise comparison of nucleotide sequence identities of NZ-2010-541 with other EV68 strains (shown in %)

**Strain**	**Accession number**	**Genome**	**5**^**′**^**UTR**	**P1**	**P2**	**P3**	**3**^**′**^**UTR**
JPOC10-378	AB601883	91.3	92.9	90.9	91.0	91.3	98.5
Fermon	AY426531	88.5	90.2	87.8	88.7	87.9	95.5
37-99	EF107098	93.4	93.0	93.2	93.4	93.4	100.0

Alignment was performed using the ClustalW algorithim in MEGA, with pairwise distance calculations based upon ungapped regions of the alignment.

*5’ UTR* = 5’ untranslated region; *P1* = precursor P1; *P2* = precursor P2; *P3* = precursor P3.

## Discussion

We have reported an emergence of EV68 infections in New Zealand in 2010. EV68 was detected in a total of fifteen specimens between March and August 2010. We also describe a full length genome sequence of a representative EV68 isolate, which contributes to the four published full genome sequences of EV68; that of the prototype Fermon, that of the French 37-99 strain and two EV68 strains that were circulating in Japan in 2010 [5,9,16]. Until recently, EV68 was only isolated rarely [2]. However in the past couple of years EV68 has been isolated with increased frequency with its isolation being reported in various countries worldwide beginning in 2009 [7-13]. The isolation of enterovirus 68 in New Zealand in 2010 is consistent with this trend. EV68 is thought to be associated with more severe lower respiratory tract infections such as bronchiolitis and pneumonia as well as causing exacerbations of asthma [5]. New Zealand EV68 patients primarily presented with bronchiolitis, cough, coryza, wheezing and the exacerbation of asthma. Eight patients were admitted to hospital although two of those patients had underlying illness. The high number of EV68 hospitalisations within a short period of time suggests that EV68 could be highly transmissible in the community and capable of causing more severe disease particularly in young children.

The majority (80.0%) of EV68 strains identified in 2010 in New Zealand were from patients of Pacific Island or Maori descent. Of note was that the other 20.0% of cases specified their ethnicity as other suggesting ethnicity other than New Zealand European, Maori, Pacific Island or Asian. Despite the over-representation of theses ethnicities being disproportionately affected by EV68 this dataset is not large enough to conclude EV68 infection is more likely to affect New Zealand Pacific Island and Maori populations but does warrant enhanced surveillance or further study.

An alternative explanation for this is the bias of hospital users, as Pacific Island or Maori ethnic groups are usually over-represented in hospital admissions for acute respiratory tract infections [24-27]. A number of factors such as nutrition and living conditions, exposure to cigarette smoke and lack of early medical care due to cost may lead to the higher hospitalisation rates among these communities [24,26,27]. New Zealand minority groups are not alone in experiencing higher hospitalisation rates. Internationally, minority groups show higher hospital admission rates for respiratory infections than those in the majority culture [28-30]. Phylogenetic analysis of the variable VP1 region of the New Zealand EV68 isolates showed that our clinical isolates grouped in one distinct phylogenetic cluster. Comparing the New Zealand EV68 VP1 nucleotide sequences with those from the Philippines, Japan and the Netherlands showed that the New Zealand isolates clustered with EV68 isolates that were detected in the Netherlands in 2010. EV68 isolates from the Netherlands clustered into two distinct phylogenetic groups; those detected in 2009 that were circulating in the community and those detected in 2010 from EV68 positive patients that required hospitalisation [12]. Additional analysis of the EV68 isolates from the Netherlands demonstrated an amino acid deletion at residue 242 in the VP1 protein in the 2010 cluster compared with the 2009 cluster. This deletion was also observed in the New Zealand isolate NZ-2010-541 that was sequenced in this study [12]. This deletion was common to both New Zealand and Netherland hospitalised patients but not to Netherland patients treated in the community and this could indicate increased virulence leading to more severe infection [12]. Further studies are required to understand the role of this deletion in relation to virulence and disease severity and confirm this hypothesis.

It was expected that a full coverage of the NZ-2010-541 genome was not achieved by high throughput sequencing, despite our anticipation that 40,000 sequence reads with an average read length of approximately 500 base pairs should provide an average coverage of approximately 2700X. We noted 17 gaps in the high throughput sequence data with less than 5X coverage which ranged in size from 3 to 180 bp, and custom sanger sequencing primers were designed to fill these gaps.

In summary, this report describes the occurrence of a rare EV68 infection associated with lower respiratory tract infection in young children in New Zealand. The national poliovirus/enterovirus surveillance system is an essential and effective tool in identifying emerging and re-emerging enterovirus outbreaks and has the potential to inform upon public health interventions. An increased awareness of EV68 may help to detect this virus with greater frequency thus providing valuable information to healthcare providers. Generating useful phylogenetic and epidemiological data may help to reveal the reason for the increased activity of this virus in 2010.

## Competing interests

The authors declare that they have no competing interest.

## Authors’ contributions

AKT participated in the design and co-ordination of the study, carried out the molecular PCR assays and wrote the manuscript. RJH participated in the design of the study, prepared a representative enterovirus strain for whole genome sequencing and assisted with sequence alignment. JW performed sequence alignment and phylogenetic analysis. MP participated in the design of the study and assisted with sequence alignment. SMcT performed initial molecular PCR assays. CJR carried out enterovirus sequencing. J-AS carried out enterovirus sequencing and assisted with sequence alignment and phylogenetic analysis. ST participated in study design and assisted with assay development. QSH participated in study design and contributed to writing of the final manuscript. All authors read and approved the final manuscript.
